# Suprapubic Hair as a Supplementary Donor Source in Body Hair Follicular Unit Extraction: A Case Report

**DOI:** 10.7759/cureus.106127

**Published:** 2026-03-30

**Authors:** Mya T Clark, Kimberley E Meathrel

**Affiliations:** 1 Research, Bespoke Skin MD, Kingston, CAN; 2 Plastic Surgery, Bespoke Skin MD, Kingston, CAN

**Keywords:** body hair transplantation, follicular unit extraction, fue hair transplant, non-scalp donor hair, suprapubic hair

## Abstract

Body hair transplantation (BHT) is a viable option for patients with limited scalp donor reserves. Common BHT donor sites include the beard, chest, and limbs, while the clinical utility of the suprapubic region remains incompletely characterized. We describe a patient who underwent a two-stage BHT procedure, using chest donor hair in the first stage and suprapubic donor hair in the second stage. Crown hair coverage increased from 67.9% at baseline to 84.6% following BHT and platelet-rich plasma (PRP) therapy. This case report contributes semi-quantitative data on an underreported BHT donor site and highlights the need for standardized patient selection criteria and comprehensive follow-up.

## Introduction

Body hair transplantation (BHT) is a hair restoration technique that extracts follicles from non-scalp body areas and can serve as a valuable adjunct for patients with significant scalp hair loss [1]. BHT follicular unit extraction (FUE) enables harvesting from a range of non-scalp sites, most commonly the beard, chest, and extremities, each with distinct growth characteristics [2]. The suprapubic region is less frequently described in contemporary BHT literature, despite its potential to provide coarse hair that may contribute to improved coverage in select patients [3].

Body hair grafts exhibit greater variability in growth characteristics and yield, typically requiring a longer maturation period (12-24 months) compared with traditional scalp grafts [4,5]. Most published studies emphasize the more common donor sites, with the beard and torso generally demonstrating the closest similarity to scalp hair [1,6-9]. When suprapubic hair is included in published reports, it is often grouped within heterogeneous body hair cohorts without isolated analysis or quantitative outcome measures [2-5]. As a result, while suprapubic hair is generally considered a less conventional donor source, its clinical utility and performance characteristics relative to other body hair donor sites remain incompletely defined.

We present a case demonstrating the deliberate use of suprapubic hair as a supplementary donor source when both chest and standard scalp donor areas were insufficient. This report includes an eight-month photographic and semi-quantitative follow-up, providing clinical insight into an underreported donor region.

## Case presentation

Patient intervention and timeline

A 49-year-old male patient of Indian descent presented for hair transplant evaluation. His BMI was 23.7 kg/m². He reported no history of smoking and no known comorbidities. Hormonal evaluation did not reveal any significant abnormalities. He first noticed crown hair thinning in 2003, which he described as progressively worsening. He reported a family history of male pattern baldness and had been taking finasteride for three to four years with minimal effect. Twelve years earlier, he underwent a crown-targeted follicular unit transplantation (FUT) at another clinic, which he considered unsuccessful due to inadequate coverage and the presence of an FUT scar. On examination, his hair loss corresponded to a Norwood V pattern. The patient's goal was to undergo FUE, with priority given to the crown and previously scarred regions.

In October 2023, the patient underwent FUE to the crown and FUT scar using a 0.9 mm punch (SmartGraft, Clarion Medical, Cambridge, Ontario, Canada) with oscillating extraction and tumescent anesthesia. A total of 2,500 grafts were transplanted, 242 of which were harvested from the chest, with the remaining grafts obtained from the scalp. Following extraction, grafts were stored in saline under hypothermic conditions on ice and were subsequently separated under microscopic magnification into single- and multi-hair follicular units prior to implantation. Recipient sites were created following standard FUE implantation techniques with attention to angulation, direction, and distribution appropriate for the crown and scarred regions.

Following the procedure, the patient underwent two platelet-rich plasma (PRP) follow-up sessions in January 2024 and November 2024. Despite excellent improvement in the FUT scar (the primary focus of the first stage), he remained dissatisfied with the crown area. With the patient’s consent and awareness of the associated risks and expectations of suprapubic donor hair, it was elected in February 2025 to supplement the transplant with suprapubic donor grafts, targeting maximum density at the vertex and tapering anteriorly toward the hairline. The procedure involved a total of 1,770 grafts harvested exclusively from the suprapubic region using the same FUE technique and instrumentation parameters. Grafts remained on ice under hypothermic storage conditions for approximately five hours between harvesting completion and final implantation. The suprapubic stage achieved an approximate implantation density in the range of ~12-18 grafts/cm², consistent with a coverage-oriented approach given donor limitations. The patient was prescribed antibiotics for the suprapubic donor area and subsequently underwent two additional PRP sessions in February 2025 and June 2025.

Follow-up and outcomes

Postoperative hair coverage was assessed using standardized Canon digital photographs (Canon Inc., Tokyo, Japan) obtained at each follow-up visit under consistent clinic lighting, camera distance, angle, and a guided patient positioning system. Images were cropped to a consistent region of interest (ROI) encompassing the transplanted area, highlighting the crown region of the scalp.

To minimize variation from image resolution differences, each photograph crop was standardized prior to analysis. Although crops were drawn to the same size on screen, variations in source image resolution could cause differences in measured area. To address this, all images were checked in ImageJ (Fiji; National Institutes of Health, Bethesda, MD, USA) using Image → Properties… to confirm uniform pixel dimensions. When discrepancies were present, images were rescaled to a consistent pixel count (e.g., 400 × 400 px) via Image → Scale… before cropping and analysis.

Photographs were then converted to eight-bit grayscale. Thresholding was applied to highlight the non-hair-bearing crown regions in red. Thresholded images were converted to binary, and the red pixel area (scalp) was quantified using Analyze → Set Measurements → Area. The total ROI area was determined by measuring the uncropped ROI.

**Figure 1:**
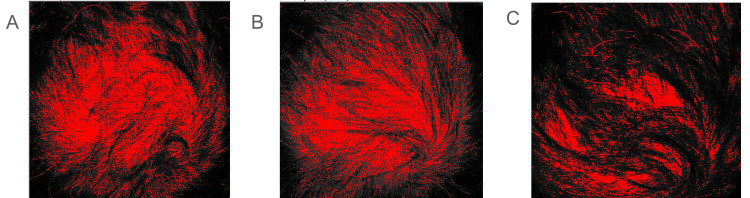
Threshold-based analysis of crown hair coverage at sequential treatment stages Red-highlighted pixels represent exposed, non–hair-bearing scalp, while non-red regions correspond to hair-covered areas. (A) Baseline image prior to intervention; (B) After first-stage transplantation using scalp and chest donor grafts; (C) After second-stage transplantation using suprapubic donor grafts.

Hair coverage percentage was calculated as $\% \text{Hair Coverage} = 100 - \left( \frac{\text{Red Area}}{\text{Total ROI Area}} \times 100 \right)$. This approach provided a semi-quantitative assessment of hair coverage over time, allowing both visual and numerical evaluation of transplanted graft integration as seen in Figures 1A-1C and Table 1, respectively. Crown hair coverage increased from 67.9% at baseline to 72.7% following the initial scalp and chest grafting procedure and further increased to 84.6% after suprapubic graft transplantation and subsequent PRP therapy.

**Table 1:** Semi-quantitative crown hair coverage analysis across treatment stages

Image ID	Threshold	Total Area (px²)	Red Area (Scalp, px²)	% Scalp (Red Area / Total × 100)	% Hair Coverage (100 - % Scalp)
Baseline	54-255	400x400 =160,000	51,439	32.1	67.9
FirstStage Scalp and Chest Approach	69-255	400x400 = 160,000	43,651	27.3	72.7
Second Stage Suprapubic Region Approach	35-255	400x400 = 160,000	24,735	15.4	84.6

## Discussion

This case demonstrates a semi-quantitative evaluation of suprapubic and chest donor hair using standardized photographic analysis, providing information on graft integration over time. 

In a large series by Umar, pubic hair was utilized in a small subset of BHT patients (n = 6, 4.9%), with a mean graft count of 823, which was modest compared with other body donor sources (trunk: 2,515; beard: 1,715). In contrast, our case achieved 1,770 suprapubic grafts. Although Umar noted slightly lower healing and growth scores for pubic hair, overall satisfaction remained high (8/10) [2]. These findings align with the modest improvement observed in our patient and reinforce the potential of suprapubic hair to enhance outcomes. 

However, the variation in reported graft yields and outcomes across donor sites highlights the inherent variability of BHT. This case, therefore, stresses the importance of setting realistic patient expectations, as results may not match those achieved with conventional scalp-to-scalp transplantation [4]. Such variability further reinforces the need for standardized patient selection criteria and scoring systems, including the Torso Donor Index [5] and the Sanusi FUE Scoring (SFS) Scale (originally developed for scalp donors but later adapted for beard and body hair assessment) [10].

Objective and quantifiable assessment methods may refine patient counseling and outcome evaluation. Technologies such as TrichoScan, which combines epiluminescence microscopy with digital image analysis, enable measurement of hair density, diameter, growth rate, and the anagen/telogen ratio [11]. By automating the labor-intensive phototrichogram process, TrichoScan makes objective evaluation more feasible in clinical practice [12]. Similarly, light-emitting diode (LED)-based photoacoustic imaging can non-invasively quantify follicle density and subdermal follicle angles, capturing both surface and subsurface features to assess follicle survival and orientation [13]. Deep-learning algorithms further advance this approach by detecting individual follicles, classifying hair counts, and calculating density from standard photographs, providing reproducible data applicable to retrospective analyses [14].

Strengths and limitations

A key strength of this approach is the ability to track progressive improvement in follicular density and visual coverage, beyond subjective aesthetic assessment or patient satisfaction scores. Limitations include the single-patient design, the relatively short follow-up period for suprapubic graft maturation, and the inherent variability in body hair characteristics. The image analysis methodology is semi-quantitative and, therefore, subject to inherent limitations associated with pixel-based measurements. In addition, PRP treatments during the follow-up period may influence the interpretation of graft-specific outcomes. 

## Conclusions

This case demonstrates that suprapubic and chest donor hair can be effectively integrated into crown hair restoration. While outcomes may vary, suprapubic hair represents a viable option for enhancing density in areas with limited scalp donor supply. Objective assessment tools and careful patient selection are essential to optimize results and set realistic expectations in BHT. 
